# Author's Response to Letter to the Editor Concerning “Glucagon‐Like Peptide Agonists for Weight Management in Antipsychotic‐Induced Weight Gain: A Systematic Review and Meta‐Analysis”

**DOI:** 10.1111/acps.13784

**Published:** 2024-12-22

**Authors:** Bea Campforts, Maarten Bak, Patrick Domen, Therese van Amelsvoort, Marjan Drukker

**Affiliations:** ^1^ Department of Psychiatry & Neuropsychology Maastricht University Maastricht The Netherlands

Dear Editor,

We would like to express our gratitude to the authors for their interest in our review and for the time they dedicated to reviewing and commenting on our work [1]. We are pleased with the underlining of the potential impact glucagon‐like peptide‐1 (GLP‐1) agonists may have on psychopathology. This represents a significant avenue for future investigation, particularly given the influence of GLP‐1 agonists on dopamine homeostasis in reward‐related brain regions. This topic has yet to be sufficiently explored. Previous trials utilising GLP‐1 agonists for the treatment of the metabolic side effects associated with antipsychotic medications have generally ignored the interaction with psychopathological changes as a potential side effect.

The authors have raised methodological concerns about our meta‐analysis [2]. Nevertheless, we believe that the arguments and assumptions made by the authors are not entirely accurate.

First, it should be noted that our inclusion criteria were explicitly defined as encompassing both randomised clinical trials (RCTs), and non‐randomised controlled trials, as well as cohort studies [2]. The rationale behind the inclusion of RCTs as well as non‐RCTs may be open to debate. However, the objective was to include as many valid trials of GLP‐1 agonists in this population as possible. Despite RCTs being regarded as the gold standard, it remains to be seen whether the populations included in RCTs are fully representative of the clinical population in mental health services. Moreover, non‐RCTs are not, by definition, inherently inferior in terms of quality or outcome status. A recent analysis by Taipale et al. estimated that only approximately 20% of patients with schizophrenia spectrum disorders may be represented in RCTs [3]. This leaves 80% of patients in this population generally excluded from RCTs. Cohort studies are often considered to have a lower level of evidence than RCTs, but the inclusion of cohort studies increases the external validity. In light of the aforementioned evidence, the decision to include non‐randomised studies is justifiable, with due consideration of the limitations of both RCTs and non‐RCTs.

Second, we do not understand why the exclusion of Prasad's case series [4] is being questioned. While the study is undoubtedly intriguing, it has to be excluded while it fails to meet the a priori defined inclusion criterion that explicitly prohibits the inclusion of studies employing multiple weight reduction interventions simultaneously. The authors explicitly indicate that the majority of patients were still using metformin at the time of the introduction of semaglutide. If the intervention with metformin was unsuccessful, why not switch to a GLP‐1 agonist, in this case semaglutide, rather than adding it? It is not possible to discern or assess the effect of semaglutide in isolation from metformin in this specific study. Therefore, it is impossible to conclude whether the weight loss achieved by these patients is solely attributable to the addition of semaglutide. As said, the study is of significant value as it is the first to investigate the use of semaglutide in this population. Nevertheless, to assess the efficacy of semaglutide, it would be essential to investigate the use of semaglutide as a standalone weight intervention in this population and compare the results with those of a suitable comparator, such as metformin. This has been done in a non‐randomised study that we recently published [5].

Third, the authors claim that the review excluded studies based on the type of antipsychotic used. However, when looking at the studies included and excluded from the review, no such exclusion was found [2]. Two studies (Maagensen et al. 2021; Ishoy et al. 2017, references 19 and 30 in the Supplement) were excluded on the grounds of the absence of data for the primary outcome (weight or BMI). As the primary objective of the review is to examine the use of GLP‐1 agonists as a weight management strategy, the exclusion of these studies is considered appropriate.

In conclusion, our review provides a comprehensive summary of the available evidence on GLP‐1 agonists for weight loss in this population to date. We agree that it is disappointing that no studies on semaglutide could be included in this review. However, several RCTs investigating the efficacy and safety of semaglutide for weight loss in this population are currently ongoing [6]. The results of these studies are awaited with interest. We believe that GLP‐1 agonists are promising interventions to counteract the metabolic effects of antipsychotics. This is, ultimately, our mutual interest and concern as clinicians and researchers.

## Conflicts of Interest

The authors declare no conflicts of interest.

## Data Availability

The authors have nothing to report.
